# Ultrafast structural dynamics of the orthorhombic distortion in the Fe-pnictide parent compound BaFe_2_As_2_

**DOI:** 10.1063/1.4947250

**Published:** 2016-04-18

**Authors:** L. Rettig, S. O. Mariager, A. Ferrer, S. Grübel, J. A. Johnson, J. Rittmann, T. Wolf, S. L. Johnson, G. Ingold, P. Beaud, U. Staub

**Affiliations:** 1 Swiss Light Source, Paul Scherrer Institut, CH-5232 Villigen PSI, Switzerland; 2 Institute for Quantum Electronics, ETH Zürich, CH-8093 Zürich, Switzerland; 3 Ecole Polytechnique Fédérale de Lausanne, Laboratoire de Spectroscopie Ultrarapide, ISIC, FSB, CH-1015 Lausanne, Switzerland; 4 Karlsruhe Institute of Technology, Institut für Festkörperphysik, D-76021 Karlsruhe, Germany; 5 SwissFEL, Paul Scherrer Institut, CH-5232 Villigen PSI, Switzerland

## Abstract

Using femtosecond time-resolved hard x-ray diffraction, we investigate the structural dynamics of the orthorhombic distortion in the Fe-pnictide parent compound BaFe_2_As_2_. The orthorhombic distortion analyzed by the transient splitting of the (1 0 3) Bragg reflection is suppressed on an initial timescale of 35 ps, which is much slower than the suppression of magnetic and nematic order. This observation demonstrates a transient state with persistent structural distortion and suppressed magnetic/nematic order which are strongly linked in thermal equilibrium. We suggest a way of quantifying the coupling between structural and nematic degrees of freedom based on the dynamics of the respective order parameters.

## I. INTRODUCTION

The close proximity of different types of order in the complex phase diagram of the Fe pnictide high-*T_C_* superconductors bears witness of the importance of electronic, magnetic, and structural degrees of freedom for their properties. Superconductivity emerges out of the antiferromagnetic ground state of the parent compounds upon doping or applying pressure,^1^ and early on, magnetic interactions were proposed to play a key role in the superconducting state.^2^ A structural transition from a high-temperature tetragonal to a low-temperature orthorhombic crystal structure occurring simultaneously with or slightly preceding the magnetic transition in temperature is also common to most of the compound families. A strong biquadratic coupling of the structural and magnetic transitions has been proposed based on the temperature behavior of the respective order parameters,^3,4^ and a nematic phase of local orbital and magnetic anisotropy has been shown to persist in the temperature range between the Néel temperature *T_N_* and the structural transition temperature *T_S_*.^5–7^ This nematic order was suggested to be the driving force of the structural transition,^7–9^ yet also a structural origin of the nematic order was discussed.^10^ A competition of superconductivity with the structural distortion was also found in some compounds.^11^ Even though a strong magneto-elastic coupling was proposed to be present in these materials and an enhanced coupling to electrons has been found recently for a relevant phonon mode,^12,13^ the exact nature of the interplay of the structural and magnetic transitions is still an open question.

Time-resolved experiments offer the opportunity to study this interplay directly and to disentangle coupled degrees of freedom on their respective time scales. Recent optical pump-probe experiments^14,15^ and experiments using time-resolved photoemission techniques^16^ have investigated the dynamics of the antiferromagnetic state of several FeAs compounds and found an ultrafast suppression of the antiferromagnetic order on the timescale of ∼100 fs, followed by a relatively fast recovery within a few ps. Similar dynamics of the magnetic order was observed by time-resolved THz spectroscopy in BaFe_2_As_2_, which also revealed a striking signature reminiscent of the antiferromagnetic phase even above the magnetic transition temperature due to the presence of a coherent phonon.^17^ The dynamics of the nematic phase was investigated using the transient anisotropy of the optical reflectivity,^18^ which also showed a fast suppression but a much slower recovery on a timescale of ∼30 ps, assigned to nematic fluctuations.

However, no conclusive investigation of the dynamics of the structural distortion has been available so far. A recent experiment reported by Gerber *et al*.^13^ investigated the dynamics of the splitting of an x-ray Bragg reflection using a 2D-detector in the orthorhombic phase of BaFe_2_As_2_ after optical excitation closely below *T_S_*. This study did not find any change of the peak splitting within the data accuracy.^13^ However, only a limited time window up to 4.5 ps was investigated in that study, and no time-resolved rocking curves were presented, which is necessary to capture the full dynamics of Bragg reflections in reciprocal space.

Here, we investigate the photoinduced dynamics of the orthorhombic to tetragonal transition in the Fe pnictide parent compound BaFe_2_As_2_ after intense optical excitation. We find an initial reduction of the peak splitting of time-resolved rocking curve scans of the tetragonal (1 0 3) reflection, which progresses on a timescale of ≈35 ps and is much slower than the suppression of magnetic and nematic order. While this transient suppression of magnetic and nematic order with persistent orthorhombic distortion demonstrates a distinctly disparate dynamics of structural and nematic degrees of freedom, we suggest a way of quantifying the coupling based on the dynamics of the energy transfer between nematic fluctuations and the lattice.

## II. EXPERIMENTAL DETAILS

**FIG. 1.**
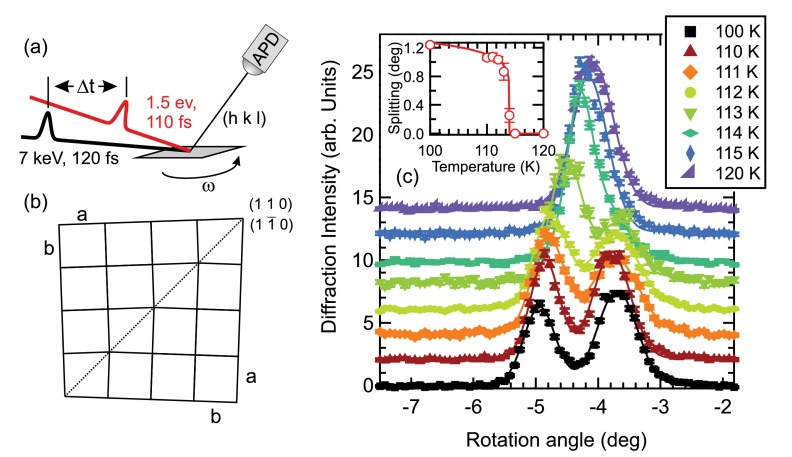
Experimental setup and orthorhombic splitting. (a) Sketch of the experimental configuration of the femtosecond time-resolved x-ray diffraction experiments. (b) Sketch of the formation of orthorhombic twin domains in the low-temperature phase. The expansion of the orthorhombic *a* axis leads to two different domain configurations, which share a common diagonal along the orthorhombic (1 1 0)/$\left(1\;\bar{1}\;0\right)$ directions. (c) Temperature-dependent x-ray rocking curves of the (1 0 3) reflection, showing a splitting into two peaks below the structural transition. Error bars are determined by the shot noise distribution, and lines are fits of two Lorentzian-squared line shapes (see text). Inset: Peak splitting Δω as a function of cryojet temperature. Error bars are 95% confidence intervals of the fittings, and the line is a power law function (see text).

The time-resolved x-ray diffraction experiments were performed at the FEMTO hard x-ray slicing facility at the Swiss Light Source^19^ in an asymmetric diffraction configuration.^20^ The experimental geometry is sketched in Fig. 1(a). The single crystal of BaFe_2_As_2_ was grown by a self-flux method^21^ and cleaved prior to the experiment to reveal a large and flat (0 0 1) oriented surface. It shows an equilibrium structural transition from tetragonal to orthorhombic structure (space group I4/mmm to *Fmmm*) at $T_{S}\approx 137\;K$, as verified by static x-ray diffraction. The temperature of the sample was controlled and stabilized between 100 and 140 K during the measurements using a cryogenic nitrogen blower. The femtosecond x-ray pulses with a pulse duration of ∼120 fs full-width at half maximum (FWHM) were incident on the sample at a grazing angle of $\alpha_{i}=0.43{}^{\circ}$, matching the x-ray penetration depth to the optical penetration depth of ∼25 nm. The x-ray energy was set to 7 keV with a bandwidth of 0.05% by a Ge(111) monochromator, yielding a photon flux at the sample position of ∼40 photons/pulse at a repetition rate of 2 kHz. The x-ray beam was tightly focused to <10 μm vertically by a Kirkpatrick-Baez (KB) mirror and weakly focussed horizontally to 300 μm. The diffracted x-ray photons were detected by a fast avalanche photodiode (APD). For time-resolved measurements, the sample was excited at a 1 kHz repetition rate by 1.55 eV laser pulses with a duration of ∼110 fs FWHM, which were incident on the sample at an angle of 10° to the surface. The overall time resolution was estimated to be ∼160 fs.

Rocking curve scans of the tetragonal (1 0 3) lattice reflection at negative pump-probe delays for a rotation about the sample surface normal (*ω* in Fig. 1(a)) are shown in Fig. 1(c) for various temperatures of the nitrogen gas jet upon heating. For temperatures <115 K, a clear splitting of the rocking curve into two peaks is observed, which is indicative of the orthorhombic phase. This peak splitting originates from the formation of twin domains along the orthorhombic (1 1 0) and $\left(1\;\bar{1}\;0\right)$ directions (tetragonal (1 0 0) and (0 1 0) directions), which leads to a slightly different orientation of the inequivalent orthorhombic *a* and *b* axis in the different domains,^22–24^ as sketched in Fig. 1(b). Note that the splitting into exactly two peaks observed here indicates the presence of one dominant kind of orthorhombic domain wall orientation in the probed spot,^22,24^ and the angle of the splitting in the rocking curves Δω is directly proportional to the orthorhombic order parameter^23^ δ=(a−b)/(a+b). To extract the splitting of the peaks, the rocking curves are fitted by two Lorentzian-squared line shapes, shown as lines in Fig. 1(c). The size of the splitting determined by the fitting is shown in the inset and quickly disappears at a temperature of ∼114−115 K, where the two peaks merge into one (tetragonal) peak. The temperature dependence of the splitting can be well described by a power law behavior with a critical exponent β≈0.12 and $T_{S}=114\;K$. While the critical exponent is in agreement with literature values,^3^ the reduced transition temperature observed here compared to the equilibrium structural and antiferromagnetic transition temperatures $T_{S}\approx T_{N}\approx 137\;K$ of BaFe_2_As_2_^25,26^ is due to an average heating effect by the pump laser pulses (absorbed fluence $F=3.3\;{\text{mJ}/\text{cm}}^{2}$) and the limited cooling power of the nitrogen gas jet. In the following, we will use this orthorhombic splitting to investigate the time-dependence after optical excitation of the orthorhombic distortion.

## III. RESULTS

**FIG. 2.**
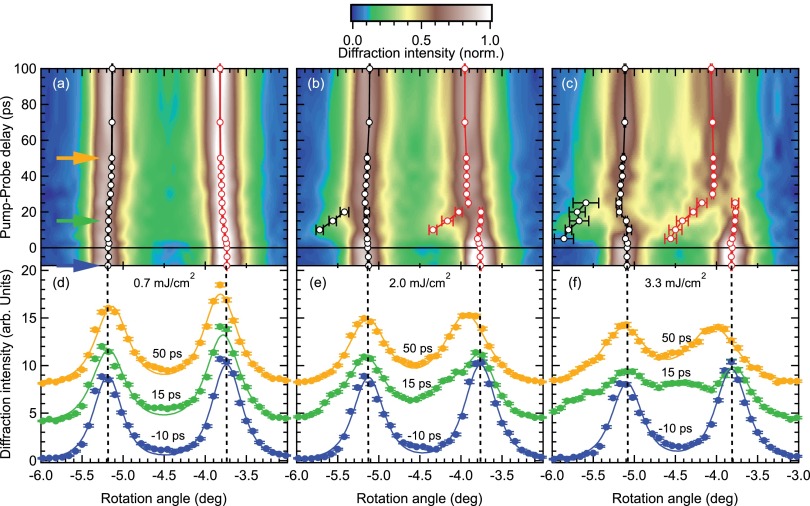
(a)–(c) Time-resolved rocking curves for various fluences as a function of rotation angle and pump-probe delay on a false colors scale. Markers denote peak positions obtained from peak fits (see text), and error bars are 95% confidence intervals. Colored arrows in (a) indicate the pump-probe delays of the rocking curves shown in (d)–(f). Note the strain-wave induced sidebands in panels (b) and (c) at early times after excitation. (d)–(f): Rocking curves (markers) and fits (lines) for selected pump-probe delays. Dashed vertical lines mark the peak positions before excitation determined from the fits. Error bars of the data are standard errors of the shot distribution.

The transient diffraction intensities of the (1 0 3) peak at a base temperature of T=100 K and as a function of sample rotation and pump-probe delay are shown in Figs. 2(a)–2(c) in a false color scale for three different absorbed pump fluences. Panels (d)–(f) show the respective rocking curve scans extracted for selected pump-probe delays, together with fitting functions used to extract the orthorhombic splitting (see below). Before excitation (blue curves in panels (d)–(f)), we observe the splitting of the rocking curve in two peaks for all fluences, similar as in Fig. 1(c). After arrival of the pump pulse at t=0 ps, for the smallest fluence a slight shift of the right peak towards the left can be seen in Fig. 2(a), which proceeds on a timescale of several 10 s of picoseconds. On the same timescale, an even smaller shift of the left peak towards the right is observed, overall leading to a slight reduction of the peak splitting, and hence the structural distortion. The peak shifts are more clearly seen in Fig. 2(d) as a shift of the peak center relative to the dashed lines, which mark the peak positions before excitation.

For higher excitation fluences exceeding $F\approx 1\;{\text{mJ}/\text{cm}}^{2}$, albeit at late times a qualitatively similar and stronger reduction of the peak splitting is observed, the dynamics at early times appear more complicated. In particular, we observe additional peaks within the first ∼20 ps, which appear at the negative rotation sides of the two split peaks and which quickly relax towards the main peaks. This becomes most apparent in the data at the highest fluence in Figs. 2(c) and 2(f). The appearance of such side-peaks is a known feature in time-resolved diffraction at high excitation levels which is due to the formation of a coherent strain wave, that is launched by the optical excitation and travels towards the bulk of the crystal.^27–30^ As the x-ray probe volume averages over strained and unstrained parts of the sample, which are separated by the strain wave front, interference of the diffracted x-rays from different depths leads to the appearance of satellite peaks, and a shift of the main peaks. The relaxation of the satellite peaks towards the main peaks and the relative sizes of the peaks are determined by the dynamics of the transient strain profile as the strain wave front moves though the probed volume,^27,29^ which depends on the elastic properties of the crystal. In addition to the strain wave, a significant peak broadening and decrease of diffraction intensity is observed after excitation, demonstrating disorder due to remaining strain and an elevated lattice temperature.

In order to quantitatively analyze the orthorhombic distortion as characterized by the peak splitting as a function of pump-probe delay, the data are fitted by a model consisting of two Lorentzian-squared line shapes. To capture the complicated behavior induced by the strain wave, two additional side-peaks have been included in the fits for 5 ps≲t≲25 ps in Figs. 2(b) and 2(c), where the distance of each main and its satellite peak (which is characteristic of the transient lattice strain) is described by a common parameter. Such a description of the strain wave is a purely phenomenological approach, and a proper model of the strain dynamics based on a realistic simulation of the transient strain profile^31^ could yield additional information.^28,29^ However, as we are mainly interested in the peak positions and their separation, the chosen model yields a good description of the data while keeping model assumptions and computational effort small. Exemplary fits are shown for selected pump-probe delays in Figs. 2(d)–2(f). The individual peak positions determined by this fitting procedure are shown as the black and red markers in Figs. 2(a)–2(c) for the left and right peak, respectively. The positions of the additional peaks used to describe the strain wave satellites are indicated by additional markers, where they have been included in the fits.

**FIG. 3.**
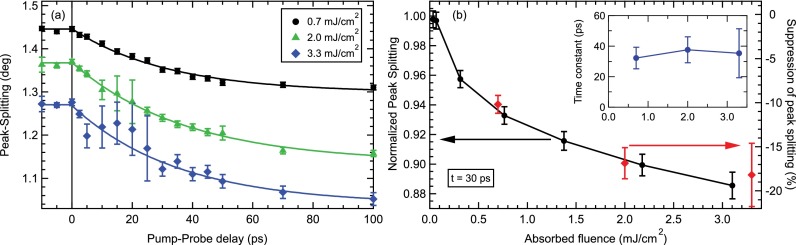
(a) Peak splitting as a function of pump-probe delay derived from the data of Fig. 2. Error bars are derived from the accuracy of the fits, and lines are exponential fits to the data (see text). (b) Normalized peak splitting at a pump-probe delay t=30 ps as a function of absorbed pump fluence (black dots). The fitted amplitudes of the peak splitting suppression (*A* in Eq. (1)) are shown as the red diamonds for comparison. Inset: Fluence dependence of the exponential time-constants of the peak splitting dynamics.

As a next step, the transient peak splitting is determined from the fits as the distance of the positions of the two peaks, which directly represents the dynamics of the orthorhombic order parameter. In the delay range, where the satellite peaks due to the strain wave were included, the area-weighted average of the respective main- and side-peak positions has been taken as an effective position for each peak. As the effects of the strain wave such as the satellite peaks and systematic peak shifts are expected to be the same for both peaks and thus do not influence their distance, this approach represents a good procedure to extract the effective peak splitting, albeit leading to larger error bars. The transient peak splittings are shown in Fig. 3(a) as a function of pump-probe delay. For all fluences, the continuous reduction of the peak splitting on a timescale of several 10 s of picoseconds is observed that was already visible in Fig. 2. The offset of the initial peak splitting before excitation for the different fluences indicates the average heating effect induced by the pump laser that reduces also the transition temperature (see Fig. 1).

To extract the timescale of the observed reduction of the orthorhombic distortion, the transient peak splitting is fitted by an exponential decay function

$$\Delta \omega (t)=\Delta \omega_{0}\left[1-\Theta (t)A(1-e^{-t/\tau })\right],$$ (1)

where Θ(t) is the Heaviside function, $\Delta \omega_{0}$ is the peak splitting before excitation, and *A* and *τ* are the amplitude and time constant of the suppression, respectively. All three data sets are well fitted by Equation (1) and yield time constants of τ≈35 ps independent of the pump fluence as shown in the inset of Fig. 3(b).

**FIG. 4.**
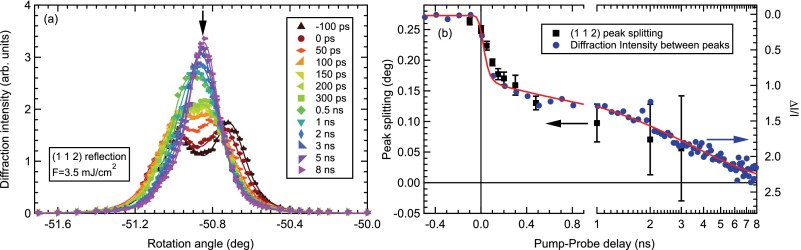
(a) Time-dependent rocking scans of the (1 1 2) reflection, for different pump-probe delays on a nanosecond timescale. Lines are fits to the data (see text) and the arrow marks the position of the time-trace shown in (b). (b) Peak splitting (black squares) of the (1 1 2) reflection obtained from fits to the data in (a), and pump-induced change of diffraction intensity ΔI/I at a fixed angle between the split peaks (blue circles). Note the inverted scale of the right-hand axis. The line is a fit of a two-timescale excitation model to the intensity data (see text).

For the dynamics at longer timescales, we investigated the transient peak splitting of the tetragonal (1 1 2) reflection, using the full picosecond bunch of the storage ring with a temporal resolution of ∼70 ps. Fig. 4(a) shows transient rocking curve scans for various delays up to several nanoseconds for an absorbed fluence of $F=3.5\;{\text{mJ}/\text{cm}}^{2}$ that reveal the complete suppression of the peak splitting and transformation into a single tetragonal (1 1 2) peak. Note that the initial splitting of the (1 1 2) peak is smaller compared to the (1 0 3) peak due to the larger in-plane component of the scattering vector. The transient peak splitting is extracted by fitting two Lorentzian-squared peaks, and shown in Fig. 4(b) as the black squares. The transient pump-induced gain of diffraction intensity ΔI/I at a fixed angle between the two split peaks (black arrow in Fig. 4(a)) is shown as the blue circles and reproduces the dynamics of the peak splitting very well. Note that the axis has been inverted to facilitate comparison with the peak splitting. Remarkably, the fast ps dynamics observed above is followed by a second, much slower dynamics that leads to the complete suppression of the peak splitting within several nanoseconds. A fit of the transient diffraction intensity by a model similar to Equation (1), but including two exponential decay functions, where one was fixed to the fast timescale $\tau_{\text{fast}}=35\;\text{ps}$ observed above, yields a slow timescale $\tau_{\text{slow}}=3.5\pm 0.6\;\text{ns}$ for the complete transition to the tetragonal state.

## IV. DISCUSSION

Remarkably, even the initial fast timescale of the suppression $\tau_{\text{fast}}\approx 35\;\text{ps}$ is much slower than the suppression of magnetic order that occurs on a 100 fs timescale, followed by a recovery on a fast ps timescale.^16,17^ However, considering the complex domain pattern of intertwined orthorhombic domains that form in the low-temperature phase offers a natural explanation of a much slower timescale of the suppression of the structural distortion compared to the dynamics of magnetic/nematic order. While the lattice constants in the vicinity of the domain boundaries can adopt to equal tetragonal sizes very quickly, deeper into the orthorhombic domains such a change involves a concerted translation of all atoms of each unit cell to adopt for the new lattice symmetry. Thus, the transformation towards a tetragonal state needs to progress from the domain boundaries towards the volume of the domains driven by acoustic phonons, and thus will be limited by the speed of sound. Indeed, considering the longitudinal sound velocity^32^ of $v_{l}\approx 6\;\text{nm}/\text{ps}$ and the orthorhombic domain size of typically 10 μm (Ref. 24) yields a time constant for the suppression in the order of 1–2 ns, very similar to the slow dynamics $\tau_{\text{slow}}$ of the full suppression of the orthorhombic splitting.

Still, the occurrence of the distinctive fast timescale $\tau_{\text{fast}}\approx 35\;\text{ps}$ cannot be explained by the slow acoustic domain dynamics. As already mentioned above, the early structural response is determined by the dynamics in the vicinity of domain boundaries, which are not limited by geometric constraints, and thus represent the intrinsic dynamics of the coupled structural and magnetic/nematic system. The observation of a transient state at very early times after excitation, where the magnetic and nematic ordering is suppressed, while the structural distortion is still unaffected, demonstrates distinctly different trajectories of the structural and magnetic/nematic order parameters in the excited state, which are strongly coupled in thermal equilibrium.^3,4^

Additional information about the strength of this coupling can potentially be gained from the timescales of equilibration of the two subsystems. Remarkably, the timescale found here for the *suppression* of structural order $\tau_{\text{fast}}$ is very similar to the 30 ps timescale of the *reformation* of nematic order observed by the transient anisotropy of the optical reflectivity,^18^ which was attributed to nematic fluctuations. A possible explanation of the similar timescales could involve the energy transfer between nematic and structural degrees of freedom in the excited system. On the same footing as nematic order is restored, the energy released from nematic fluctuations leads to the reduction of the orthorhombic distortion near the domain boundaries, where atomic rearrangements on a fast ps scale are feasible. In such a scenario, the observed timescales can provide valuable information about the couplings of nematic and structural degrees of freedom in the complex potential energy surface of the coupled magnetic, nematic, and structural system,^8,9^ similar to the two-temperature model, which describes the energy flow between electronic and lattice degrees of freedom.^33^

Finally, we address the fluence-dependence of the pump-induced reduction of the peak splitting. Fig. 3(b) displays the relative suppression amplitudes of the fits in Fig. 3(a) (red diamonds), as well as the normalized peak splitting at a fixed pump-probe delay of t=30 ps (black circles) as a function of absorbed pump fluence. The suppression of the peak splitting shows an initially fast increase with fluence, which becomes much slower above a critical fluence of $F_{\text{crit}}∼0.2-0.3\;{\text{mJ}/\text{cm}}^{2}$. This corresponds to a transient lattice temperature after electron-lattice equilibration of the excited near-surface layer of $T_{\text{lat}}\approx 150\;K$, estimated from the lattice heat capacity.^34^ Whereas the fast increase of response reflects the energy required to heat the crystal to the structural transition temperature *T_S_*, the slower increase above $F_{\text{crit}}$ can potentially be associated with enhanced nematic fluctuations, leading to a stronger driving force for the initial suppression of structural distortion. In addition, the energy transported into the crystal by the strain wave could play a role in the fluence dependence.

## V. CONCLUSION

In conclusion, we investigated the structural dynamics of the orthorhombic distortion in BaFe_2_As_2_. The suppression of the orthorhombic splitting is characterized by two distinctive timescales, where the initial fast reduction is characterized by a timescale of ≈35 ps, followed by a much slower dynamics on a nanosecond timescale. While the slow nanosecond dynamics are consistent with the geometric constraints of the orthorhombic domain arrangement that limits a complete suppression of the orthorhombic distortion to the sound velocity, the faster timescale represents the intrinsic dynamics of the system close to domain boundaries, which are still much slower than the suppression of magnetic and nematic order. The dynamics of the respective order parameters in such a transient state with suppressed magnetic and nematic order and persistent structural distortion offer a route to quantify their coupling directly in the time domain.
